# Effect of mode of delivery on postpartum health-related quality of life

**DOI:** 10.1590/1806-9282.20231003

**Published:** 2024-03-25

**Authors:** Esra Keles, Leyla Kaya, Neşe Yakşi, Zahide Kaya

**Affiliations:** 1 University of Health Sciences Turkey, Kartal Lütfi Kırdar City Hospital, Department of Gynecologic Oncology – İstanbul, Turkey.; 2 University of Health Sciences Turkey, Zeynep Kamil Women and Children’s Disease Training and Research Hospital, Department of Obstetrics and Gynecology – İstanbul, Turkey.; 3 Amasya University, School of Medicine, Department of Public Health – Amasya, Turkey.; 4 Uskudar State Hospital, Internal Medicine Clinic – İstanbul, Turkey.

**Keywords:** Cesarean section, Episiotomy, Delivery, obstetric, Pregnancy, Quality of life

## Abstract

**OBJECTIVE:**

The aim of the study was to explore the impact of mode of delivery on health-related quality of life in mothers.

**METHODS:**

This cross-sectional study was conducted between May and August 2022 on healthy singleton pregnant women aged between 18 and 45 years. Data on socio-demographic variables, clinic features, pregnancy and birth characteristics, and neonatal outcomes were collected. Health-related quality of life was assessed by using EQ-5D-5L questionnaire.

**RESULTS:**

A total of 1,015 healthy pregnant women were included. The EQ-5D-5L index score was higher in those with regular sleep patterns (p<0.001), those who did physical activity (PA) during pregnancy (p<0.001), those who received spousal support (p<0.001), and those with very good and good perceived health (p<0.001). EQ-5D-5L index and EQ-5D-5L-VAS scores were lower in those with unplanned pregnancy, those who preferred cesarean section, those who had cesarean section, those who underwent episiotomy, and those who admitted to the intensive care unit (p<0.001). Emergency cesarean section and elective cesarean section had the lowest and second lowest health-related quality of life mean scores, while normal vaginal deliveries had the highest health-related quality of life mean scores, respectively (p<0.001).

**CONCLUSION:**

This study showed that health-related quality of life was higher after vaginal delivery than after cesarean section. In addition, spousal support, regular sleep pattern, and PA during pregnancy play an important role in maternal health-related quality of life.

## INTRODUCTION

Pregnancy, delivery, and puerperium are important periods that affect women physically, mentally, and socially and cause considerable changes in their quality of life (QoL). During postpartum period, the mother needs to recover and get used to her new roles and responsibilities^1^. While puerperal changes usually resolve within 6 weeks following delivery, many women suffer from postpartum complications for a prolonged time^2^. Postpartum recovery is of paramount importance as it affects the QoL of both the mother and the newborn^3^.

In recent years, the rate of cesarean sections (CS) has increased globally. By 2030, there will be some countries with this rate over 60%. World Health Organization (WHO) has warned about the growing trend in CS and recommends countries to maintain a 10–15% rate^4^. In 2017, the overall delivery rate of CS in Turkey was 51.2%^5^. The majority of mothers still prefer CS over vaginal deliveries, despite studies demonstrating that cesareans can result in a number of complications. It appears that pregnant women lack awareness regarding the consequences of delivery methods^6^. Thus, it is imperative to apprise them regarding the advantages and disadvantages of cesarean and vaginal deliveries.

Health-related quality of life (HRQoL) has been accepted as a valid indicator of maternal health^7^. A thorough understanding of the impact of delivery methods on pregnant women’s HRQoL is critical in order to design and implement effective health interventions for this unique group. Despite the widespread use of the EQ-5D-5L questionnaire in different populations and diseases, there is an inadequate understanding of HRQoL assessment in pregnant women in Turkey.

The number of CS is escalating as more women are electing to have the procedure. To the best of our knowledge, there is a lack of data regarding the effects of delivery mode on HRQoL among Turkish pregnant women. Therefore, this study aims to fill this research gap in the literature by investigating the impact of the mode of delivery on HRQoL in postpartum women using a preference-based HRQoL measure.

## METHODS

This cross-sectional study was carried out in the obstetric unit of a tertiary health facility, between May and August 2022. The institution is a public hospital that has received accreditation under the International Baby Friendly Hospital Initiative, developed by WHO and United Nations Children’s Fund. It provides maternity and child health services at no cost and is the largest tertiary healthcare facility in Istanbul. The present study obtained permission from the EuroQol Research Foundation and approval from the Research Ethics Committee (Approval date: 06.04.2022 number: 49). The study adheres to the principles of the Helsinki Declaration. All subjects provided informed consent prior to data collection.

Participants were healthy singleton pregnant women aged between 18 and 45 years, greater than 28 weeks of gestation, literate, and willing to participate in the study. Exclusion criteria were (1) mothers with chronic medical conditions (pre-eclampsia, diabetes, chronic hypertension, asthma, gestational diabetes mellitus, cholestasis); (2) with risk of preterm birth, placenta previa, myoma uteri, polyhydramnios, oligohydramnios, multiple gestations; (3) under 18 years old or over 45 years old; (4) had a depression/psychiatric disease; (5) had given birth to a baby with anomalies; (6) had given birth to a baby with health problems (intrauterine growth restriction, etc.); and (7) had issues that were stressful such as death of a loved one, divorce, or family disruptions.

Data on socio-demographic variables, clinic features, pregnancy and birth characteristics, and neonatal outcomes were collected. HRQoL was assessed by using EQ-5D-5L questionnaire. The EQ-5D-5L is a two-part instrument. In the first part, the EQ-5D-5L instrument includes five different health dimensions: mobility, self-care, usual activities, pain or discomfort, and anxiety or depression. The severity levels of each dimension are rated on a scale of 1 (no problems) to 5 (extreme problems). The second part of the questionnaire includes EQ-VAS, a self-rating on a 20-cm vertical scale in which 0 and 100 indicate the worst and best imaginable health statutes. A higher score indicates lower quality of life. As recommended by the EuroQol Research Foundation, the EQ-5D-5L utility values presented were derived from the United Kingdom (UK) value sets, due to the lack of country-specific data for Turkey^8^.

### Statistical analysis

The data collected in the study were transferred to the Epi info 7.2 program and analyzed. Numbers, percentages, median values, and minimum and maximum values are used to describe descriptive characteristics. The data were tested for normality using Kolmogorov-Smirnov tests. Chi-square test for two categorical variables, Mann Whitney U test for pairwise comparisons, and Kruskal-Wallis test for continuous variables were performed. The relationship between two continuous variables was evaluated with the Spearman correlation test. A p-value was set at 0.05 in order to determine the level of statistical significance.

## RESULTS

Out of total deliveries conducted, 588 (57.9%) were vaginal deliveries, 193 (19.0%) were elective CS, 201 (19.8%) were emergency CS, and 33 (3.3%) were instrumental deliveries. It was found that 178 (17.5%) pregnant women participated in vigorous-intensity, 235 (23.2%) moderate-intensity, and 602 (59.3%) light-intensity physical activity (PA).

In total, 902 babies (88.9%) did not receive noninvasive respiratory support, while 113 babies (11.1%) received. Notably, 60 (5.9%) babies were admitted to neonatal intensive care unit (NICU), and 955 (94.1%) were not admitted to NICU.

The EQ-5D-5L index and VAS scores were higher in those who had a regular sleep pattern (p<0.001), those who did PA during pregnancy (p<0.001), those who received spousal support (p<0.001), and those with very good and good perceived health (p<0.001) (Table 1).

**Table 1:** Association of EQ-VAS and EQ5D index score with demographic, social, and clinical variables

		EQ5D Index Score		EQ-VAS	
		Median (min–max)	p-value*	Median (min–max)	p-value*
Cigarette smoking	Yes	-0.59 (-0.59–1.00)	0.112	80 (75–85)	0.570
	No	0.57 (-0.59–1.00)		75 (35–100)	
Sleep pattern	Regular	0.59 (-0.59–1.00)	**<0.001**	80 (35–100)	**<0.001**
	Irregular	0.03 (-0.59–1.00)		70 (40–100)	
Physical exercise during pregnancy	Regular	0.59 (-0.59–1.00)	**<0.001** ^§^	85 (50–100)	**<0.001** ^§^
	Irregular	0.59 (-0.59–1.00)		80 (40–100)	
	None	0.08 (-0.59–1.00)		70 (35–100)	
Spousal support during pregnancy and birth	Yes	0.59 (-0.59–1.00)	**<0.001**	80 (35–100)	**<0.001**
	No	-0.08 (-0.59–1.00)		65 (40–90)	
Perceived status of health	Very Good	0.65 (-0.59–1.00)	**<0.001** ^§^	90 (80–100)	**<0.001** ^§^
	Good	0.59 (-0.59–1.00)		80 (40–100)	
	Fair	0.04 (-0.59–1.00)		65 (40–90)	
	Poor	-0.59 (-0.59–1.00)		55 (35–85)	
	Very Poor	0.52 (-0.59–1.00)		65 (40–100)	

*Mann-Whitney U–test.

^§^Kruskal-Wallis test. Statistically significant values are denoted in bold.

EQ-5D-5L index and EQ-5D-5L-VAS scores were lower in those with unplanned pregnancy, those who preferred CS, those who had CS, those who underwent episiotomy, and those who were admitted to the intensive care unit (ICU) (p<0.001) (Table 2).

**Table 2:** Association of EQ-VAS and EQ5D index score with obstetric and reproductive health-related characteristics.

		EQ5D Index Score		EQ-VAS	
		Median (min–max)	p-value*	Median (min–max)	p-value*
Intention of pregnancy	Planned	0.58 (-0.59–1.00)	**0.004**	80 (40–100)	**<0.001**
	Unplanned	0.04 (-0.59–1.00)		70 (35–95)	
Mode of delivery Preferences	Normal vaginal delivery	0.59 (-0.59–1.00)	**<0.001**	80 (35–100)	**<0.001**
	Cesarean section	0.04 (-0.59–1.00)		70 (40–100)	
Mode of delivery	Normal vaginal delivery	0.88(-0.59–1.00)	**<0.001** ^§^	85 (40–100)	**<0.001** ^§^
	Instrumental NVD	0.30 (0.04–0.85)		80 (55–90)	
	Elective cesarean	0.04 (-0.59–0.52)		60 (35–85)	
	Emergency cesarean	-0.59 (-0.59–1.00)		60 (40–90)	
Episiotomy during birth	Yes	0.59 (-0.59–1.00)	**<0.001**	85 (50–100)	**<0.001**
	No	0.10 (-0.59–1.00)		70 (35–100)	
Perineal tear during birth	Yes	0.36 (0.04–0.59)	**<0.001**	80 (55–90)	0.648
	No	1.00 (-0.59–1.00)		75 (35–100)	
Degree of perineal tear	No	1.00 (-0.59–1.00)	**<0.001** ^§^	75 (35–100)	0.890^§^
	1st	–		–	
	2nd	0.35 (0.04–0.59)		80 (55–90)	
	3rd	0.54 (0.54–0.54)		80 (80–80)	
Need of blood transfusion	Yes	0.59 (-0.59–0.65)	0.976	60 (50–75)	**0.001**
	No	0.56 (-0.59–1.00)		80 (35–100)	
Maternal admission to ICU	Yes	0.04 (-0.59–1.00)	**0.014**	50 (40–70)	**<0.001**
	No	0.58 (-0.59–1.00)		80 (35–100)	

*Mann-Whitney U–test.

^§^Kruskal-Wallis test. NICU: Neonatal intensive care unit; NVD: normal vaginal delivery. Statistically significant values are denoted in bold.

Mothers whose newborns required respiratory support or who were hospitalized in the ICU had lower EQ-5D-5L index and EQ-5D-5L-VAS scores.

Emergency CS and elective CS had the lowest and second lowest HRQoL mean scores, while normal vaginal deliveries had the highest HRQoL mean scores, respectively (p<0.001) (Table 3).

**Table 3:** Association of EQ5D health dimensions with mode of delivery.

	Mode of delivery								p-value*
	NVD		Instrumental NVD		Elective CS		Emergency CS		
	Mean	SD	Mean	SD	Mean	SD	Mean	SD	
EQ5D-mobility	1.59	0.74	2.82	0.58	4.19	0.68	4.78	0.54	**<0.001**
EQ5D-self care	1.59	0.74	2.82	0.64	4.20	0.67	4.79	0.53	**<0.001**
EQ5D-usual activities	1.61	0.77	2.97	0.68	4.20	0.67	4.82	0.52	**<0.001**
EQ5D-pain/discomfort	1.62	0.78	2.79	0.60	4.20	0.67	4.80	0.53	**<0.001**
EQ5D-anxiety/depression	1.76	1.08	3.82	0.92	4.19	0.70	4.77	0.66	**<0.001**

*Kruskal-Wallis test. NVD: normal vaginal delivery; CS: cesarean section. Statistically significant values are denoted in bold.

## DISCUSSION

This study found that PA during pregnancy, sleeping regularly, receiving spousal support, and having good perceived health were associated with higher HRQoL scores. Significant poorer EQ-5D-5L index scores were found in women who had unplanned pregnancies, those who preferred CS, those who had a CS, those who underwent episiotomy, and those who were admitted to ICU. In addition, having a meconium-contaminated newborn, the newborn being admitted to the ICU, and noninvasive respiratory support for the newborn were linked to a lower EQ5DL index score.

This study indicated a considerable difference in HRQoL by birth mode. According to the HRQoL scores, spontaneous vaginal births were the highest, followed by instrument-assisted vaginal births, elective cesareans, and emergency cesareans, respectively. The study findings are in accordance with several studies that show HRQoL improved after vaginal delivery in the early postpartum period and 5 years after delivery^9^. In addition, they concur with a recent review indicating that a CS negatively affected HRQoL^10^. However, not all studies agreed, some showed that CS does not contribute to poor QoL, and others showed no significant difference between delivery methods^11^. The discrepancy between the literature can be attributed to the different study methodologies, such as the instruments used for measuring QoL and the location of studies.

Our study revealed that gestational age serves as a predisposing factor for improved HRQoL, which is contrary to Martínez-Galiano et al.’s findings^7^ that gestational age was a risk factor associated with reduced HRQoL.

Our findings were similar to those of Martínez-Galiano et al.^7^, which showed that perineal tears and episiotomies were related to poor postpartum HRQoL, whereas other studies failed to demonstrate such an association^12^. Nevertheless, their studies did not differentiate between different types of perineal lesions as our study did, but did take into account more severe perineal lesions that cause more discomfort^13^.

Regular exercise during pregnancy has positive effects on physical and mental health of mothers. Comparison of our findings with those of other studies confirmed that PA during pregnancy is associated with improved HRQoL^14^. On the contrary, a study conducted in Iran found no association between PA in pregnancy and HRQoL^15^. A possible explanation for this might be the high prevalence of physical inactivity among Iranian pregnant women.

Following a regular sleep pattern was observed to have a positive effect on postpartum QoL in our research, which is congruent with other studies^16^. In the same vein, a recent review has provided evidence that poor sleep quality was linked to a lower HRQoL during pregnancy^17^.

Spousal support was ascertained as a factor that augmented the QoL of pregnant women, which is in agreement with other studies^18^. Therefore, it can be inferred that partner support may have a positive effect on gestational HRQoL.

Maternal preference for CS was another factor contibuting to a worse postpartum QoL in our study, which overlapped with earlier studies, which found that compared with women who plan to give birth vaginally, those who request a CS reported less perceived postpartum HRQoL^19^. According to a previously published study^20^, women opting for CS have difficulty in preparing themselves for motherhood before deciding on such a procedure, which may explain why their health is poor during pregnancy.

Admission of newborn to NICU was identified as a contributor to reduced QoL among mothers, which is in line with the study by Rai and Rani^21^. In a longitudinal study, it was shown that admission of newborn to NICU may be related to poor maternal QoL up to 12 months^22^.

### Limitations

There are several caveats that must be borne in mind. First, we were unable to examine the impact of factors that influence the relationship between mode of delivery and postpartum HRQoL in the long term. Second, since the study was conducted in a developing country, the results may not be applicable to all settings. Notwithstanding these limitations, this study has advantages, including large sample size and utilization of a widely used preference-based HRQoL measure. To the best of our knowledge, the present study is one of the most comprehensive assessments of HRQoL and modes of delivery in Turkish pregnant women.

## CONCLUSION

This study showed that HRQoL was higher after vaginal delivery than after CS. In addition, spousal support, regular sleep pattern, and PA during pregnancy play an important role in maternal HRQoL. Policymakers must translate this information into healthcare policies to improve maternal HRQoL.

## Data Availability

The dataset used and/or analyzed in the study is available from the corresponding author on reasonable request.
